# Drying characteristics of faecal sludge from different on-site sanitation facilities

**DOI:** 10.1016/j.jenvman.2020.110267

**Published:** 2020-05-01

**Authors:** Samuel Getahun, Santiago Septien, Jaime Mata, Tosin Somorin, Ian Mabbett, Christopher Buckley

**Affiliations:** a Pollution Research Group, University of KwaZulu-Natal, Durban, 4041, South Africa; b Offshore Renewable Energy Engineering Centre, Cranfield University, Cranfield, MK43 0AL, UK; c Chemistry Department, Swansea University Prifysgol Abertawe, Bay Campus, Swansea, SAl 8EN, United Kingdom

**Keywords:** Drying, Faecal sludge, Valorisation, Temperature, Heat of drying, Calorific value

## Abstract

Drying is one of the treatment techniques used for the dual purpose of safe disposal and energy recovery of faecal sludge (FS). Limited data are available regarding the FS drying process. In this paper the drying properties of FS were investigated using samples from ventilated improved pit (VIP) latrines and urine diversion dry toilets (UDDT) and an anaerobic baffle reactor (ABR) from a decentralized wastewater treatment systems. Moisture content, total solids content, volatile solids content, water activity, coupled thermogravimetry & differential thermal analysis (TGA-DTA) and calorific value tests were used to characterize FS drying. Drying kinetics and water activity measured at different moisture content during drying (100 °C) were similar for the samples from different on-site sanitation facilities. Experimental heat of drying results revealed that FS requires two to three times that of the latent heat of vaporization of water for drying. Drying temperature was more significant than the sludge source in determining the final volatile solids content of the dried samples. This was reinforced by the dynamic TGA that showed considerable thermal degradation (2–11% dry solid mass) near 200 °C. Below 200 C, the calorific value of the dried samples exhibited no significant difference. The average calorific values of VIP, UDDT and ABR samples at 100 °C were 14.78, 15.70, 17.26 MJ/kg dry solid, respectively. This suggests that the fuel value of FS from the aforementioned sanitation facilities will not be significantly affected by drying temperature below 200 °C. Based on this study, the most suitable temperature for drying of FS for a solid fuel application was found to be 150 °C.

## Highlights

• Poorly managed faecal sludge has a direct impact on the environment and human health.
• Thermal drying characteristics of faecal sludge from different onsite sanitation facilities was studied.
• The source of the faecal sludge samples had little effect on drying kinetics.
• Temperature was the critical parameter that affected the volatile solids content and calorific value of the dried material.
• Suitable temperature to disinfect and dry faecal sludge for a solid fuel use was identified.

## 1 Introduction

World Health Organisation (WHO) has reported an estimated 4.2 billion people globally lacked safely managed sanitation facilities in 2017 (WHO, 2019). In developed countries, the toilets, sewer, and wastewater treatment systems require significant amount of land, energy and water, and are expensive to build, maintain and operate. Existing alternatives that are less expensive are often unappealing because they do not kill disease-causing pathogens, have impractical design, or retain odours and attract insects. The global sanitation community has made great efforts in trying to resolve sanitation problem. One of the major challenges facing this quest is finding effective and sustainable faecal sludge (FS) management techniques for the disposal of the sludge emptied from the full pits (Strande and Brdjanovic, 2014).

Since 2011, transformative sanitation technologies that function without connection to external water source, energy or sewer systems have appeared. The two main innovative transformative proposals, Reinvent the Toilet Challenge (RTTC) and Omni-processor system, were supported by the Bill & Melinda Gates Foundation (BMGF, 2012). One of the next generation technologies that came through the RTTC is the Nano-Membrane Toilet. This toilet uses a membrane to remove the water from human waste and leaves solids that can be utilized as fuel or fertilizer (Hanak et al., 2016, Onabanjo et al., 2016). The Omni-processor is an eco-friendly system that turns faeces to water and dry waste by boiling the sludge. Potable water is produced by condensing and filtering the vaporised water. The system uses the steam power generated by burning the dried sludge. Most of these systems involve drying and efficient resource recovery.

Due to its high nutrient and organic matter content FS has the potential to be used as a soil conditioner and fuel source (Gold et al., 2017, Muspratt et al., 2014, Strande and Brdjanovic, 2014). Its application as soil conditioner has been tested and showed a good result (Bai et al., 2018, Fumasoli et al., 2016, Mnkeni and Austin, 2009, Cofie et al., 2005). The viability of FS as fuel has been demonstrated at a laboratory and pilot scale (Gold et al., 2017), however it has not yet been implemented in industry. It looks promising based on the use of wastewater treatment sludge as an alternative fuel in the cement industry in Europe and the US (Usón et al., 2013, Boesch and Hellweg, 2010). According to several studies the average calorific value of FS is 17 MJ/kg dry solids (Muspratt et al., 2014), which is comparable to the 8.0–23 MJ/kg dry solids observed with bio-solids (Modinger and Mayr, 2006, Spinosa and Vesilind, 2001, Vesilind, 2001). This means direct economic benefits could emerge from FS with the correct resource recovery technology that would in turn help ensure sustainable provision of adequate sanitation (Andriessen et al., 2019, Bassan et al., 2013; Murray and Ray, 2010).

Thermal drying is one of the oldest and ubiquitous unit operation applied to remove the moisture and sanitize the FS (Niwagaba et al., 2014). The moisture in FS can be as high as 99% (wet basis) (Chen et al., 2002) and drying can reduce moisture to below 5% dry solid (Leonard et al., 2004), consequently, reducing the cost for handling and transport. In addition, drying FS at temperature ≥80 °C stabilizes the material and also inactivates pathogens such as Ascaries eggs if exposed for 5 s or more (Naidoo et al., 2019). Drying concentrates the energy in the sludge by removing water and increases the calorific value, consequently transform the sludge into an acceptable combustible material. Information of moisture distribution within sludge and understanding the bond strength (boundedness) of the moisture to the solid are vital for the selection of optimal dewatering and drying methods (Deng et al., 2011). Several factors such as humidity, air velocity, sludge origin and the physical transformations of the material during the process affect the drying operation, temperature is the most influential parameter. Temperature affects the drying time (Banga and Singh, 1994), rheological properties (Septien et al., 2018, Woolley et al., 2014a, Woolley et al., 2014b), thermal stability (Devi and Saroha, 2015, Vesilind and Ramsey, 1996) and the physicochemical characteristics of the end product (dried sludge) such as calorific value and nutrient content (Septien et al., 2020, Tao et al., 2008, Seginer et al., 2007).

Previous researchers showed the potential of FS as a fuel source (Seck et al., 2015, Muspratt et al., 2014, Skjeggerud et al., 2009, Modinger and Mayr, 2006, Spinosa and Vesilind, 2001). The characteristics of dried sludge could vary depending on the drying parameters (Vesilind and Ramsey, 1996). Up until now, no literature was found on the effect of temperature on the characteristics of FS. This study describes the effect of drying temperature and sample source on FS drying behaviour (kinetics, drying heat energy, water activity) and thermal stability (volatile solids, calorific value and thermo-gravimetric analysis). The water activity analysis was used to estimate the amount of free/bounded moisture and the potential growth of pathogens on the dried material. The effect of temperature on the drying characteristics of samples from Urine Diversion Dry Toilet (UDDT), Ventilated Improved Pit (VIP) latrines and Anaerobic Baffled Reactors (ABR) sources were evaluated.

## 2 Material and methods

### 2.1 Feedstock description

**Fig. 1:**
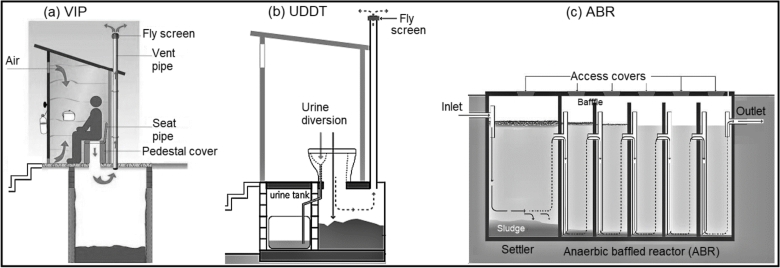
Illustrations of, (a) Ventilated Improved Pit (VIP) latrine, (b) Urine Diversion Dry Toilet (UDDT) (Strande and Brdjanovic, 2014), and (c) Anaerobic Baffled Reactors (ABR) (Tilley, 2014).

For this study, three types of FS samples were used. These were collected from VIP, UDDT and ABR facilities in eThekwini Municipality, Durban, South Africa (Fig. 1). A VIP latrine is a sealed pit fitted with a vent pipe with a fly screen on top, which facilitates airflow within the pit, restricts flies movement and thus removal of bad odour (Zuma et al., 2015, Bakare, 2014) (Fig. 1a). This latrine is used by one or more families. According to the eThekwini municipality, normally VIP pits are emptied every five years (Zuma et al., 2015). The pit is normally composed of excreta and urine but contain household trash. The VIP sample used in this study was collected from a vacuum truck after pit emptying. The sample is assumed to be homogenous and represent the entire pit. The sample was unusually wet for the general Durban VIP sludge due to the addition of water and mixing to increase its ability to be pumped (Septien et al., 2018).

The UDDT separates and collect faeces and urine in separate compartments without flush water (Fig. 1b) (Mkhize et al., 2017). Therefore it is mainly composed of excreta but contains high amount of sand that is used as a cover material to promote dry conditions within the faeces vault. The UDDT vaults are emptied every two years (Zuma et al., 2015). The vault is equipped with access door at the back of the pit that makes sample collection relatively easy. Samples from this vault were collected from the top, middle and bottom using a shovel. Large household waste (clothing, sanitary material, paper, etc.) found in the sludge were removed during sample collection.

ABR systems are used for the treatment of blackwater and greywater from nearby households and communal ablution toilets (Fig. 1c) (Reynaud and Buckley, 2015a, Reynaud and Buckley, 2015b). Accumulated scum and sludge in ABRs are emptied after two to three years when sludge-blankets have reached a height of about 1 m (Reynaud et al., 2015b). The ABR at Frasers informal settlement (Durban, South Africa) was composed of a settling tank followed by six ABR compartments (Pillay et al., 2014, Ferraz et al., 2009). Samples for this study were sucked from the settling tank by a vacuum truck and transferred to a bucket.

All the samples were collected in plastic bag within a plastic buckets with lids. After filling, the bag was knotted & the bucket lid was closed. The samples were transported to the laboratory and were stored at 4 °C in a cold room until use in order to minimize sample degradation and moisture loss. The samples were screened to remove non-homogeneous debris/trash larger than 5 mm. General physiochemical properties of the sludge, such as chemical oxygen demand (COD), moisture content, and orthophosphate content, were measured as part of sample preparation (Table 1). The average moisture content of the VIP, UDDT and ABR samples were 95, 70 and 88%, respectively. The sludge from UDDT has the lowest moisture content and a paste-like texture compared to the ABR and VIP samples, which are in a liquid form. The moisture content of the UDDT and ABR samples were in the expected range as reported by Niwagaba et al. (2014), however the VIP sample exhibited higher moisture content than what is found in Zuma et al. (2015), which is around 80%. This could be attributed to the pit emptying method used where liquid was introduced into the pit.

**Table 1:** Characteristics of the faecal sludge samples.

Sample type	Moisture content [% mass water/mass wet sample]	COD [mass O_2_/mg wet sample]	Ortho-phosphate [mg P mg wet sample]	pH
VIP	95	0.099	0.003	7.43
UDDT	70	0.409	0.005	8.13
ABR	88	0.094	0.0001	6.93

The Standard Operating Procedures (SOP) from Pollution Research Group (PRG) laboratory were used for all laboratory analysis (PRG (Pollution Research Group), 2014). The SOPs were adapted from the Standard Methods for Examination of Water and Wastewater (APHA, 2012) by this laboratory for faecal matter.

### 2.2 Moisture content and total solids analysis

The drying behaviour of samples was evaluated at 100 °C using a Moisture Analyser (*PCE-MB Series)* (APHA, 2012). Sludge samples of ~1.5 g spread on a 90 mm diameter aluminium pan were introduced into the balance of the analyser. The mass of the sample was recorded at a 1 min intervals until the change in mass was less than 1.6% per minute. Moisture ratio (MR) was used to compare the evolution of moisture loss with time (Banco-Cano et al., 2016). MR is defined as;

(1) $$MR_{\left(t\right)}=\frac{\left(M_{\left(t\right)}-M_{f}\right)}{\left(M_{0}-M_{f}\right)}$$

where $M_{\left(t\right)}$ is the moisture content (wet basis) at time t, $M_{f}$ is the final moisture content and $M_{o}$ is the initial moisture content.

The total solids of samples were determined by the mass of the samples before and after in an oven at 105 °C for a period of 24 h. The initial mass of the sample was ~20 g. The dried samples were allowed to cool in a desiccator prior to weighing.

### 2.3 Characterization of dried sludge

Four temperatures, namely, 50, 105, 150, and 200 °C, were selected to evaluate the effect of drying temperature on the material. For a better consistency, the temperature 105 °C will be referred as 100 °C in the rest of the manuscript. These temperatures were chosen to include industrially applied temperatures (100–160 °C) for similar material (sewage sludge) (Horttanainen et al., 2017) and to cover a wider range of the experimental conditions (50 & 200 °C) set apart by ~50 °C. Different analyses were performed in the dried samples, i.e.: (i) water activity (ii) ash content/volatile solids analysis; (iii) calorific value.

#### 2.3.1 Water activity measurement

Water activity was used to estimate the degree of boundedness of moisture to the solid in the sludge (Jarboui et al., 2009, Reid et al., 2007). The samples were dried in the moisture analyser to achieve various final moisture content. The water activity of samples with different moisture content was measured at 25 °C using a pre-calibrated analyser (*AquaLab Tunable Diode Laser-TDL*, METER Group, Inc. USA). For optimal performance and accuracy, periodic verification against known verification standards (unsaturated salt solutions provided by the manufacturer) was done to ensure accuracy.

#### 2.3.2 Volatile solids and ash content analysis

For the volatile solids and ash content analysis tests, ~20 g samples were weighed and ignited using a clean crucible at 550 °C for 1 h in a muffle furnace. The volatile solids and ash content of the samples were then deduced from the residual mass of sample after ignition.

#### 2.3.3 Calorific value test

Calorific value of samples were determined using Parr Bomb Calorimeter (*6200 Isoperibol*), based on the heat released by the material after oxidation with pure oxygen. Benzoic acid tablets (1 g) and ethylene glycol (0.2160 g, with a heat of combustion of 16.87 MJ/kg) were used for standardisation and as a combustion aid for wet samples, respectively. For all tests, oxygen gas pressure was set to ~3 MPa. A mass of 0.5–0.7 g per sample was used for each experiment.

### 2.4 TG-DTA tests

Experiments in a thermogravimetry analyser (TGA), coupled with a differential thermal analyser (DTA), were conducted using a *DTG-60A* from SHIMADU instruments with an operating temperature range of 25–1100 °C. This device has an accuracy of ±0.001 mg. A 70 ± 0.5 mg sample was used for each test. Samples were placed on an aluminium crucible (6 mm diameter and 5 mm height) and weighed. Unlike the tests done on the moisture analyser the samples cannot be spread to a thin layer. Experiments were conducted at constant temperature (isothermal mode) and varying temperature (dynamic mode), for each type of sludge. The isothermal tests were used to measure the drying kinetics and heat of drying at 50, 100, 150 and 200 °C. On the other hand, the dynamic tests were done in order to characterize the thermal degradation of the samples.

For the isothermal tests, the samples were heated at a constant rate of 50 °C/min whereas for the dynamic tests, they were heated at a constant rate of 5 °C/min from ambient temperature to 500 °C. The airflow rate was kept constant at 50 mL/min in both cases. The drying kinetics was expressed using the moisture ratio with respect to time. In addition, the DTA data was used to estimate the heat related to FS drying during the isothermal tests. The heat absorbed by FS during its drying was measured from the moment the mass of the sample started to decrease (beginning of drying) until the mass was stabilized (end of drying). The specific heat of drying was determined by dividing the heat measured by the device to the mass of moisture evaporated during the selected period. All the tests in this experimental study were done in triplicate. The standard error (SE) was used to show the deviation with respect to the average value of the parameter under consideration.

## 3 Results and discussions

### 3.1 Drying characteristics

#### 3.1.1 Final moisture content

VIP, UDDT and ABR samples were dried in an oven for 24 h at 50, 100, 150 and 200 °C to evaluate the effect of drying temperature. The moisture in all the samples dried at temperatures ≥100 °C were found to be completely dry when tested using the Standard Methods for the Examination of Water and Wastewater (APHA, 2012). However, the final moisture content of the sludge dried at 50 °C was 6–8%. The majority of this water was assumed to be strongly bounded. Therefore, the heat energy at this temperature was not enough to break the bound water from the capillaries of the material. This moisture was taken into account when calculating the volatile solids content and calorific values of the samples.

#### 3.1.2 Kinetics

**Fig. 2:**
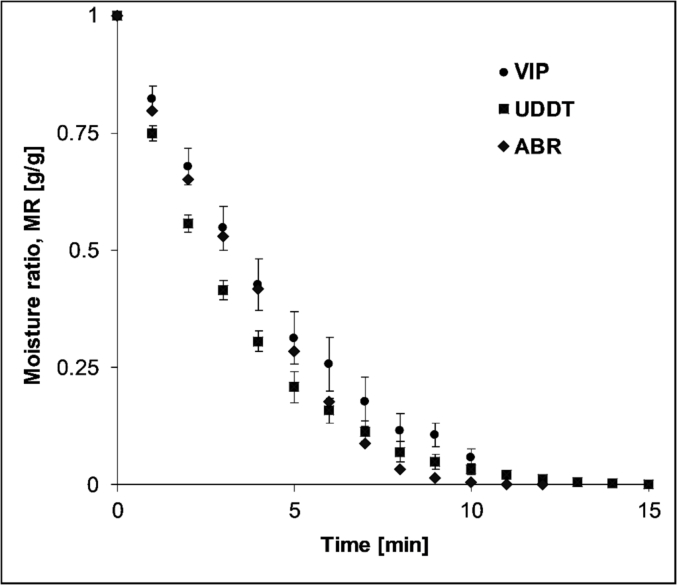
Drying curves of VIP, UDDT and ABR samples during tests in the moisture analyser.

Fig. 2 depicts the change in moisture ratio of the samples as a function of time during the tests in the moisture analyser at 100 °C. The UDDT samples dried slightly faster for the first 5 min, however this difference became insignificant after this period. The difference in moisture ratio between the samples fell under the error bar for most part of the drying period. As shown in Fig. 2, all the samples reached equilibrium almost at the same time even though they contain different amount of moisture. This could be due to high drying rate of the samples that contain high amount of free water at this temperature. These result imply that the source of the sample has little effect on the drying behaviour of the FS sample under the explored conditions.

**Fig. 3:**
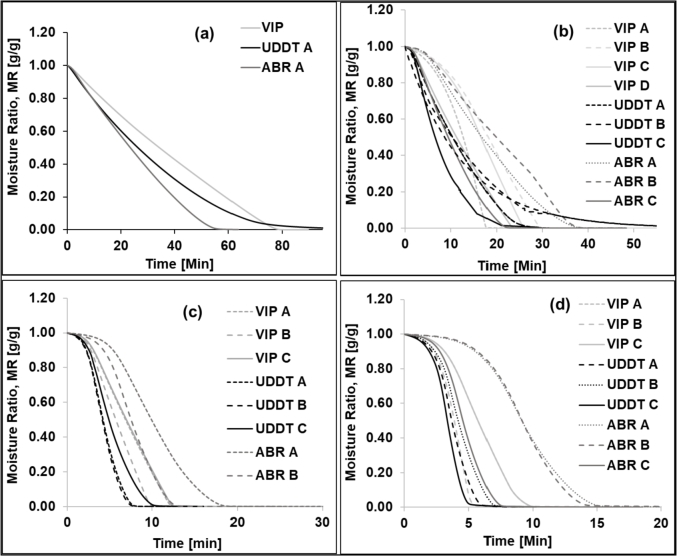
Evolution of the moisture ratio during drying of the VIP, UDDT and ABR samples at temperatures of (a) 50, (b) 100, (c) 150 and (d) 200 °C (d) during TGA experiments in isothermal mode.

Furthermore, drying kinetics of FS were evaluated using TGA tests at temperatures of 50, 100, 150 and 200 °C (Fig. 3). The plot included all the replications of each test (except at 50 °C - one test per sample was completed at this temperature) and it can be observed how significantly heterogeneity of the samples affect the kinetics. The UDDT samples exhibited better consistency and the heterogeneity was more pronounced in the samples with high moisture content (VIP and ABR). The drying time of the replicated samples varied from 1 min for the UD sample to 10 min for the ABR samples.

**Fig. 4:**
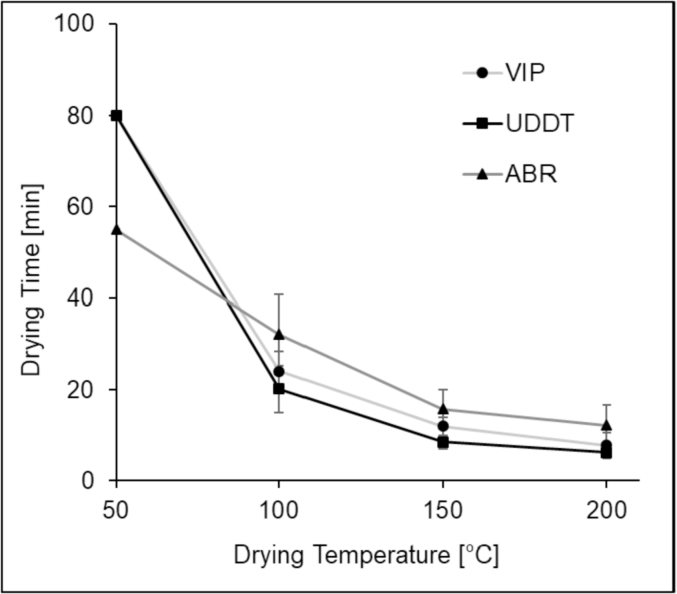
Drying time as a function of temperature for the VIP, UDDT and ABR samples during TGA analysis.

The drying time was determined from the drying curves and was plotted as a function of temperature in Fig. 4. This parameter corresponds to the period that took the mass of the FS sample to reach equilibrium (until the change in mass was lower than 1 mg/min). It was noticed that as the temperature increased, the drying time decreased for all samples. Indeed, the time for complete drying seemed to decrease exponentially as the drying temperature was increased from 50 to 150 °C (Fig. 4) but the decrease rate slowed down at 200 °C. For example, the average difference in drying time between 150 and 200 °C was only 3 min as compared to 40 min between 50 and 100 °C. As shown in Fig. 3a, b, c & d, the drying kinetics curves of the different samples exhibited some differences without a particular trend. The replication of the experiments showed a significant difference in the drying behaviour. These random differences could be attributed to the heterogeneity of the FS samples and the small amount of sample used for TGA analysis.

When the three samples were compared with respect to the drying temperature, a particular difference was observed at 50 °C. At this temperature, the ABR sample took only two-third of the time required for drying compared to the UDDT and VIP sludge (Fig. 4). The difference of drying time between these samples however diminished or disappeared as the drying temperature increased further. Note that there was a significant difference in drying time using a moisture analyser at 100 °C (Fig. 2) and TGA at 100 °C (Fig. 3b). Samples in the moisture analyser required half the time to dry in comparison to the samples in the TGA. This may be due to the difference in the operating conditions of each of the analysers (mode of heating, energy input, ventilation rate, amount of sample, surface area).

#### 3.1.3 Water activity

**Fig. 5:**
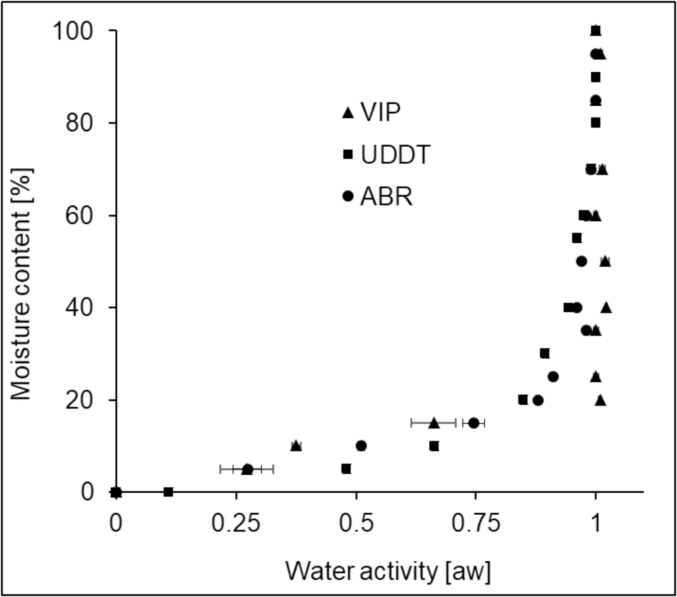
Moisture content vs. water activity (a_w_) of FS samples at different moisture contents.

Moisture can be bounded biologically, chemically or physically. Water activity ($a_{w}$) of a material can be used as a good indicator of the degree of water boundedness. High water activity (close to 1) indicates the presence of free water and, on the other hand, low water activity (close to 0) indicates strong boundedness. The water activity as a function of moisture content was shown on Fig. 5. The results clearly showed that water activity decreased with the decrease in moisture content. There was no obvious difference in water activity among samples from different source. The water activity of the samples was close to one (a_w_≈1) until the moisture content of the samples were below 60%. A significant change in the water activity took place when the moisture content was below 30%. At this point the water activity dropped to 0.85. From this point the moisture in the sludge starts to be quite bounded. These values indicate the FS samples contain more unbound water when the moisture content is greater than 30% and this implies easier drying. However, as the moisture content decrease further, the remaining water in the sludge is proportionally higher in bound and more difficult and requires higher energy to remove. To apply mechanical dewatering technology the water activity has to be close to one and the highest efficiencies obtained with wastewater sludge were around 40% dry solid (Olivier et al., 2015, Lee et al., 2006). Therefore, according to Fig. 5 mechanical dewatering process could only be effective above 60% moisture content.

In addition to this, water activity can be used to predict the potential pathogen growth on materials. Pathogenic bacteria cannot grow below a_w_ of 0.85–0.86, whereas yeast and moulds are more tolerant to a reduced water activity of 0.80 (Sagar and Kumar, 2010). Usually no growth of pathogenic bacteria nor yeast and mould occurs below a_w_ of about 0.62 (Sablani, 2006). Therefore, microbial growth and pathogenic bacteria are unlikely events for all the samples with less than 10% and 20% moisture content, respectively.

#### 3.1.4 Heat for drying

The amount of heat required to remove water from sludge is expected to be higher than for pure water. This is due to additional energy required to break the bond between the bound water and the sludge. Table 2 showed the amount of heat for drying with respect to sludge type and drying temperature. The heat required to dry the samples for most of the cases was similar and the differences were within the margins of the uncertainty, except for the VIP sample at 50 °C that recorded the minimum heat of drying (2.6 ± 0.2 MJ/kg of moisture removed). Although VIP sample recorded the minimum heat of drying at 50 °C, there was no significant variation between the three types of samples dried at other drying temperatures (100, 150 and 200 °C). Similarly heat required to vaporize water decreases from 2.4 MJ/kg to 2.3 MJ/kg when the temperature increase from 50 to 100 °C, this indicates drying temperature has little effect on the energy required to remove the water. In the temperature range, 100–200 °C, it was noted that UDDT samples have slightly higher heat of drying values than VIP and ABR samples, however the heat of drying for most of the cases falls under the same standard error margin.

**Table 2:** Amount of heat absorbed during drying at different temperatures for different types of sludge and water.

Temperature [°C]	Heat of Drying [MJ/kg water removed]			Heat of Vaporization [MJ/kg]
	VIP	UDDT	ABR	Water
50	2.6 ± 0.2	3.0 ± 0.1	4.0 ± 0.5	2.4
100	4.1 ± 0.5	5.7 ± 0.6	4.8 ± 0.4	2.3
150	3.7 ± 0.1	6.1 ± 0.1	2.4 ± 0.3	2.1
200	4.3 ± 0.9	5.6 ± 0.1	4.9 ± 0.6	1.9

On the other hand, comparison of samples from different sources showed no particular trend. VIP samples showed a slightly lower heat of drying at 50 °C, however the values at the other drying temperatures showed that the sample source has no or little effect on the heat of drying. However, it was observed that the heat required for drying was two to three times the heat of vaporization of water (Table 2) and this result can have implications on the design process of drying systems such as drying beds.

### 3.2 Thermal decomposition

#### 3.2.1 Volatile solids and ash content

The volatile solids content of the sludge varied depending on the initial organic matter content of the sample (Table 3). For all the samples dried at temperature between 50 and 150 °C there was no statistically significant loss in volatile solids content as the drying temperature increased. However, the volatile solids content of the samples dried at 200 °C decreased significantly compared to the other temperatures. The maximum and minimum volatile solids content of, VIP, UDDT and ABR samples were 0.66, 0.57 and 0.76 g/g dry mass and 0.46, 0.52, 0.63 g/g dry mass, respectively. We can then assume that drying temperature lower than 150 °C preserved the organic matter, whereas at higher temperature, the volatile solids content decreased due to burning/charring. The volatile solids content decrease, when temperature increased from 150 to 200 °C, was approximately 20, 10 and 15% for 150–200 °C for UDDT, VIP and ABR samples, respectively. With this experiment it was not possible to know the exact temperature, between 150 and 200 °C, at which the volatile solids content started to decrease. This was captured with TGA dynamic test presented in the following section.

**Table 3:** Volatile solids content of the UDDT, VIP and ABR samples after drying at different temperatures.

Type	Drying Temperature [ °C]	50	100	150	200
**UDDT**	Volatile solids (g/g dry sample)	0.59 ± 0.02	0.58 ± 0.05	0.57 ± 0.01	0.46 ± 0.02
	Ash content (g/g dry sample)	0.41 ± 0.02	0.42 ± 0.05	0.43 ± 0.01	0.56 ± 0.02
**VIP**	Volatile solids (g/g dry sample)	0.66 ± 0.17	0.64 ± 0.02	0.59 ± 0.02	0.52 ± 0.02
	Ash content (g/g dry sample)	0.34 ± 0.17	0.36 ± 0.02	0.41 ± 0.02	0.48 ± 0.02
**ABR**	Volatile solids (g/g dry sample)	0.76 ± 0.01	0.77 ± 0.04	0.73 ± 0.01	0.63 ± 0.04
	Ash content (g/g dry sample)	0.24 ± 0.01	0.22 ± 0.04	0.27 ± 0.01	0.37 ± 0.04

**Fig. 6:**
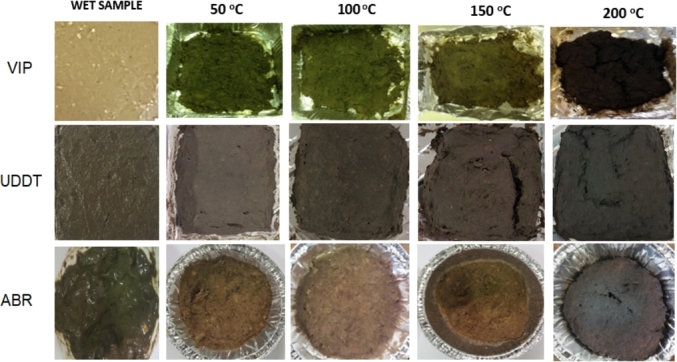
Visual observation of VIP, ABR and UDDT samples after drying at different temperatures.

From visual observation of the dried samples as the drying temperature increased, the colour of the dried samples became darker (Fig. 6), which may indicate a certain level of thermal degradation. For example, each of the samples dried at 200 °C had the darkest colour, were brittle and recorded the lowest volatile solids content.

#### 3.2.2 TGA analysis

**Fig. 7:**
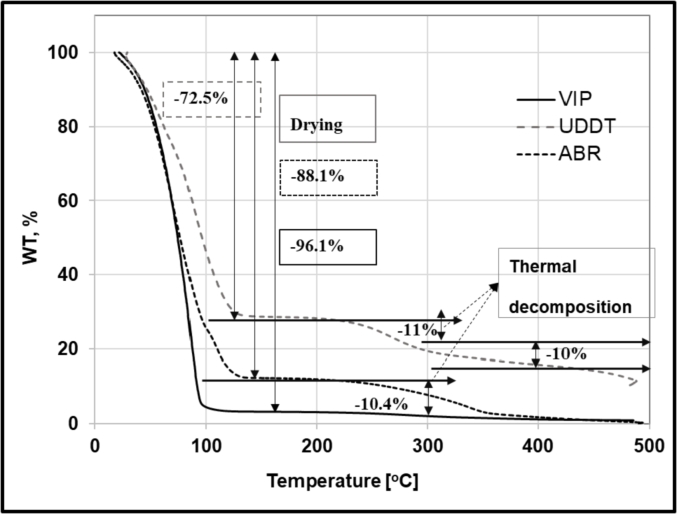
Evolution of FS samples weight as the drying temperature increases from ambient to 500 °C at 5 °C/min during dynamic tests in the TGA.

In section 3.1.2, TGA was used to evaluate FS drying kinetics at isothermal conditions, whereas in this section, the thermal stability of FS samples were evaluated using the TGA (dynamic test) as the drying temperature increased from room temperature to 500 °C. Fig. 7 depicts the evolution of sample weight as the temperature increased at 5 °C/min to 500 °C and with a constant 50 mL/min airflow rate. During this process a two stage weight loss was observed. In the first stage, the weight of the samples decreased by 96.1, 72.5, and 88.1% as the temperature increased to 115 °C for the VIP, UDDT and ABR sludge, respectively. In this stage, the weight loss was probably due to the removal of moisture from the sludge. The recorded mass losses were close to the actual moisture content of the raw sludge. The second stage took place between 250 and 350 °C where the weight of the samples decreased further by around 10, 2 and 10% for UDDT, VIP and ABR samples, respectively. This weight loss can be ascribed to thermal decomposition of the material. At this level of temperature, the sludge should decompose chemically as the heat breaks chemical bonds from the dry skeleton. This is similar to the reports of Serio et al. (2016) for canine faeces (200–250 °C), which the authors assumed to have a similar characteristics to fresh human faeces. From this dynamic TGA test it was seen thermal degradation takes effect around 200 °C which is also in agreement with the results from the previous section.

#### 3.2.3 Calorific value

**Fig. 8:**
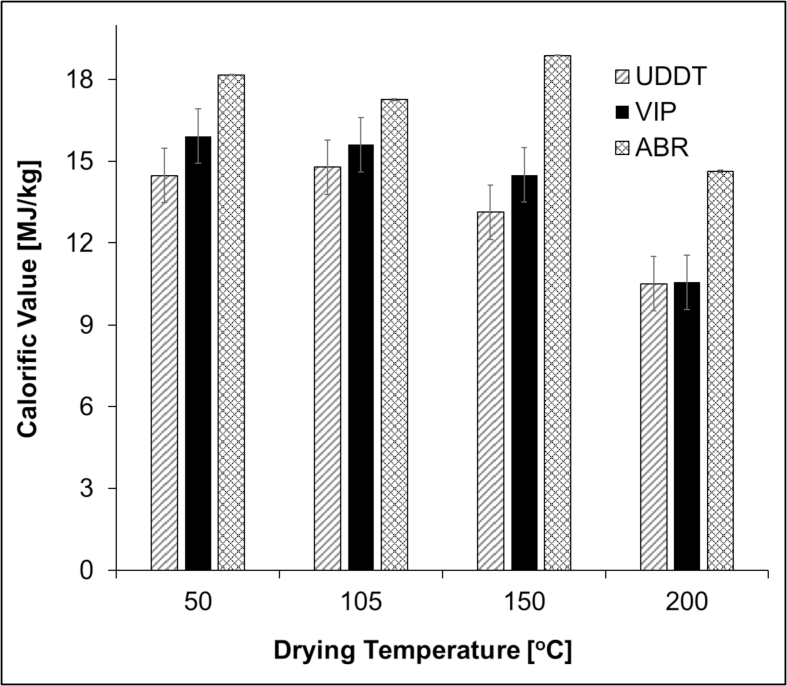
Calorific value of samples dried at 50, 100, 150 and 200 °C. The error bars show the standard error of the triplicated tests.

Calorific value of the dried sludge is a critical parameter in terms of using the material as a fuel source. One of the parameters that affect this property is drying temperature. The calorific value of the samples showed no considerable variation as the drying temperature was increased from 50 to 150 °C (Fig. 8). In this temperature range, the calorific value was approximately 15 MJ/kg for the VIP and UDDT samples and 18 MJ/kg for the ABR samples. However, further increment of the drying temperature to 200 °C resulted in a calorific value loss of approximately 30% for the UDDT and VIP samples and 20% for the ABR samples. As discussed in the previous section, a considerable amount of solid was lost due volatile solids loss as a result of thermal degradation as the temperature reached 200 °C. Hence, the decrease in the calorific value can be related to the loss in volatile solids content at this temperature. The results in this paper are similar to the work from other authors where the calorific value of FS collected from pit latrines were reported to range between 11 and 18 MJ/kg dry solid (Zuma et al., 2015, Muspratt et al., 2014) and around 25 MJ/kg for fresh faeces (Onabanjo et al., 2016, Muspratt et al., 2014).

## 4 Conclusion

The results discussed in this paper provide insights on FS drying and provide information that could be applied to improve drying technologies. The drying kinetics of samples from different sources were similar. Drying time decrease exponentially as drying temperature was increased from 50 to 150 °C. However, this pattern diminished as drying temperature was further increased to 200 °C.

Like the drying kinetics, the evolution of water activity during drying also showed a similar trend for the different sample sources. Above 60% moisture content the water activity was close to 1, which indicates the predominance of unbound water. From this, it can be assumed that the FS could be mechanically dewatered to 60% moisture content, at most. There was a rapid decrease in water activity when the moisture content reached 30%, which indicated that the remaining water was quite bounded. Further drying, to <10% moisture content, ensured water activity <0.62, which indicated the unsuitability of the environment for bacteria and pathogen growth.

The bound water in the sludge requires a higher energy input to break the bonds to remove the water. The energy required for FS drying was estimated to be two to three times that of the latent heat of vaporization of pure water. No pattern was found with respect to drying temperature. But it was noticed that UDDT samples appeared to require slightly higher heat of drying than VIP and ABR samples. These results have implications on drying and dewatering process selection and design.

One of the critical factors during drying was temperature as it affected the volatile solids content, hence the calorific value of the end product. This was reflected as the drying temperature increased from 150 to 200 °C, during which the volatile solids content and calorific value of sludge samples decreased by approximately 10–30%. This was confirmed with the dynamic tests in the TGA that showed the thermal degradation took place around 200 °C. At the other temperatures (50, 100 and 150 °C), the calorific value of the dried samples have showed no significant difference. Therefore, the most suitable temperature for drying of FS for a solid fuel application was found to be 150 °C as it dries the sludge almost as fast as at 200 °C and does not lead to thermal degradation. The source of the FS had different effects on the drying characteristics. It had little or no influence on the drying kinetics and, water activity. However, volatile solids content and calorific value were mostly influenced by the source of the sample.

## Credit author statement

**Samuel Getahun**: Carried Out Most of the Experiment and Wrote the Manuscript. **Jaime Mata**: Carried Out Some of the Experiment. **Santiago Septien:** Concieved the Orginal Idea, Provided Critical Feedback and Supervised the Project. **Tosin Somorin** and **Ian Mabbet**: Involved in the Planning of the Project. **Christopher Buckley**: Conceived the Original Idea, Provided critical Feedback, Editing and Supervision.
